# Pretransplant Desensitization of Donor-Specific Anti-HLA Antibodies with Plasmapheresis and Immunoglobulin Produces Equivalent Outcomes to Patients with No Donor Specific Antibodies in Haploidentical Hematopoietic Cell Transplant

**DOI:** 10.21203/rs.3.rs-3832106/v1

**Published:** 2024-01-08

**Authors:** Hunter Cochran, Michael Slade, Feng Gao, Sonia Godbole, Aaron Pruitt, Elisa De Togni, Chang Liu, Brenda Grossman, Ramzi Abboud

**Affiliations:** Washington University School of Medicine; Washington University in St. Louis School of Medicine; Washington University; Washington University School of Medicine; Washington University School of Medicine; Washington University in St. Louis School of Medicine; Washington University School of Medicine; Washington University in St. Louis; Washington University

## Abstract

In patients requiring haploidentical hematopoietic cell transplant (haplo-HCT), the presence of donor specific anti-HLA antibodies (DSAs) is associated with high rates of primary graft failure and poor overall survival (OS). There is limited data regarding the effect of desensitization. Adult patients undergoing haplo-HCT at Washington University School of Medicine from 2009–2021 were identified. Patients were divided into three cohorts: no DSA, untreated DSA or treated DSA. DSA testing was performed. Desensitization therapy using plasmapheresis and IVIg (immunoglobulin) was performed. We retrospectively identified 304 patients for study inclusion. 14 of 30 patients with DSAs underwent desensitization. By day +2, 57% of patients cleared all DSAs. OS was expectedly worse in patients with untreated DSAs. There were similar results between treated DSA and patients without DSA (OS median: control: 352 days vs. treated: 1331 days vs. untreated: 137 days, p = 0.02). RFS was also significantly different between the groups however with similar RFS in treated DSA and control groups (RFS median: control: 248 vs. treated: 322 v. untreated: 119, p = 0.03). Desensitization before haplo-HCT produces similar outcomes to patients without DSAs. While the optimal desensitization protocol has not been established, all patients received a backbone of plasmapheresis and immunoglobulin.

## Introduction

In many hematologic malignancies, hematopoietic cell transplant (HCT) is the only curative option. For patients who require HCTs, the use of matched sibling donors is associated with improved outcomes and is the preferred choice (1). For patients without a matched sibling, a matched unrelated donor is often available through the registry. However, registry matched probabilities vary widely by ethnicity (2). The likelihood of finding a fully matched unrelated donor for patients of European descent is 75%, compared to those of African descent, where the probability of finding a full matched donor is less than 20%. In those without a fully matched donor available, HLA mismatched HCTs between relatives, or haploidentical HCTs, have become a potential life-saving option (1). More recently, haploidentical HCTs have expanded the availability of allo-HCT, as nearly all patients have an available haploidentical donor. As nearly every patient has a living parent, one HLA haplotype matched sibling or child available, haploidentical donors are almost universally available regardless of race or ethnicity.

The presence of pre-existing antibodies to donor human leukocyte antigens (HLA) (i.e donor-specific antibodies or DSAs) is an increasingly recognized impediment to successful use of HLA-mismatched donors (3). Several recent retrospective studies have shown that the presence of DSAs leads to worse overall survival and higher rate of graft failure when compared to patients who do not have DSAs (4) (5). Other studies have shown a DSA value > 5000 MFI is correlated with worse outcomes (6) (7). However, in the absence of another suitable donor, haplo-HCTs with DSAs are sometimes necessary, and the optimal peri-transplant management in these patients is unclear (3). Most prior experience with the management of DSAs comes from the solid-organ transplant setting. Studies have shown depleting DSAs through a desensitization protocol improves outcomes after solid organ transplant (8). Although there is limited data regarding specific methods of desensitization, one solid organ kidney transplant study used plasmapheresis and IVIG with success in decreasing patients’ DSA levels to weak or negative (8). DSA levels are measured using mean fluorescence intensity (MFI). MFI of 2000 or less is considered weak (9) or clinically insignificant. This study concluded patients should be desensitized to weak or negative levels to provide the patient the best opportunity for successful transplantation (9). Another recent study shows promising evidence using double filter plasmapheresis twice in the week prior to transplant followed by a dose of rituximab in haplo-HCTs with DSAs (L. Liu et al., 2023). Data on desensitization in the HCT setting is limited, especially comparative data for outcomes for post-desensitization compared to contemporaneously transplanted patients either without DSAs or untreated DSAs.

Consequently, we performed this single center retrospective study to describe desensitization protocols at a high-volume transplant center and determine the impact of DSAs on outcomes with and without desensitization compared to a contemporaneous population of haplo-HCT patients. The goal of this study is to provide a baseline for future prospective trials of desensitization protocols in this setting.

## Methods

### Patients

Patients aged ≥ 18 and older who underwent haploidentical HCT with post-transplant cyclophosphamide (PTCy) at Washington University School of Medicine between July 2009 to July 2021 were retrospectively reviewed for inclusion. Patients were included regardless of prior transplant. Patients with unknown DSA status were excluded. Patients were divided into three cohorts based on DSA status and treatment: no DSA, untreated DSA or DSA with desensitization therapy. This study was approved by the Washington University Human Research Protections Office.

### Identifying Donor Specific Antibodies

Anti-HLA antibody testing was performed using the LABScreen Single Antigen Bead assay (One Lambda, West Hills, CA) with pretreatment by 6% EDTA (Sigma-Aldrich, St. Louis, MO) to remove complement interference (11). A mean fluorescence intensity (MFI) of 2000 was used as the cutoff for clinical relevance (9). Antibody screen results were compared with donor HLA typing (HLA-A, B, C, DR, DP, and DQ) to identify DSAs. For donor alleles not represented in the single antigen bead (SAB) panel, DSA is reported based on beads coated with the closest antigen–determined using the HistoCheck program (12). Testing was performed either prospectively after November 2014, when routine DSA screening was added to our standard pre-transplant workup, or, in cases before routine DSA screen, retrospectively using banked sera when available.

### Treatment

**Figure 1:**
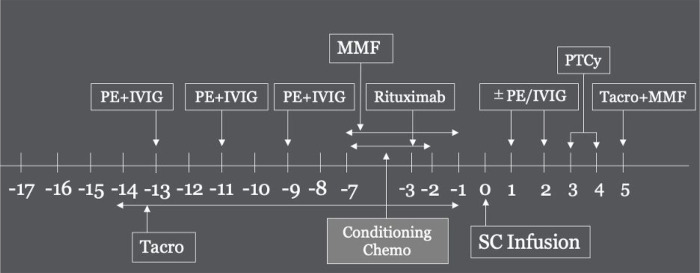
The timeline of our desensitization protocol backbone. Additional sessions added per provider discretion prior to day −13. PE plasmapheresis. IVIG Intravenous immunoglobulin. Tacro Tacrolimus. MMF Mycophenolate Mofetil. SC Infusion Stem Cell infusion. PTCy Post-Transplant Cyclophosphamide. Conditioning Chemo is dependent on provider choice and patient condition.

At our institution, we began desensitization for patients with DSA in 2014. Patients with DSA prior to 2014 were not desensitized and make up the untreated DSA group. Desensitization therapy was carried out using our standard institutional protocol of plasmapheresis and low dose intravenous immunoglobulin, adapted from Gladstone et al (9). The standard protocol included tacrolimus 1 mg IV daily starting on approximately day −14 and mycophenolate mofetil starting on approximately day −7 and continued through day −1. In addition, patients received alternate day single volume plasmapheresis on days −13, −11, and −9 followed by 100 mg/kg intravenous immunoglobulin (IVIg). Additional sessions of plasmapheresis with IVIg were performed on days + 1 and + 2 after transplant if DSA level measured on day −1 remained > 2 000 MFI. As this was not done in a prospective, controlled trial, there were variations in the actual desensitization each patient underwent. The most variation was observed in the exact days and total number of plasmapheresis/IVIg sessions performed (Table 2). In addition, one patient received rituximab 375 mg/m2 on day −3. All patients received standard GVHD prophylaxis with post-transplant cyclophosphamide on days + 3 and + 4 as well as tacrolimus and mycophenolate mofetil (starting on day + 5). See visual of our protocol in Fig. 1.

### Definitions

Clinically relevant DSAs are defined as having an MFI level > 2000 (13). Overall survival (OS) was defined as the time from diagnosis to time of death from any cause. Relapse-free survival (RFS) was defined as the time from diagnosis to disease relapse or death from any cause. Relapse was defined as > 5% blasts in bone marrow or peripheral blood. Acute and chronic graft-versus host disease (aGVHD and cGVHD, respectively) were graded according to the Keystone and National Institutes of Health criteria, respectively (14)(15). All patients were prospectively evaluated using the Karnosfky Performance Status (KPS) score and the hematopoietic cell transplantation–specific comorbidity index (HCT-CI) (16). Neutrophil engraftment was defined as the first of 3 consecutive days of absolute neutrophil count > 500 cells/mm3 after post-transplant nadir. Platelet engraftment was defined as the first of 3 measurements within 7 days showing > 20,000 platelets/mm3 without platelet transfusion in previous 7 days. Disease risk index (DRI) was retrospectively assigned to each patient in accordance with previously published work (17–19). Primary graft failure was defined as no evidence of engraftment of hematological recovery of donor cells within the first month after transplant, without evidence of disease relapse. Secondary graft failure is the loss of a prior functional graft, leading to cytopenia in at least two blood cell lines [below]. Delayed engraftment was defined as severe cytopenia of at least two cell lines and/or transfusion requirement in presence of hypoplastic/aplastic BM with full donor chimerism, in absence of severe GVHD or relapse. Disease was considered de novo if the patient had never previously received a diagnosis of MDS or received leukemogenic therapy. Active disease at transplant was defined as evidence of extramedullary disease or > 5% blasts in bone marrow or peripheral blood.

### Statistical Methods

Patient demographics and disease characteristics were summarized using counts and frequencies for categorical variables or means and standard deviations for continuous variables. Patients with negative DSA testing were used as a control group for this analysis. The distributions of patient demographics and disease characteristics across 3 cohorts (control, DSA without desensitization therapy, DSA with desensitization therapy) were compared using the Student’s t-test, Chi-square test, or Mann-Whitney rank-sum test as appropriate. The distributions of OS and RFS across DSA cohorts were described using Kaplan-Meier product limit methods and compared by the log-rank test. Cumulative incidences of relapse, treatment-related mortality (TRM), aGVHD, cGVHD, neutrophil engraftment, and platelet engraftment were estimated using Gray’s sub-distribution regression to account for competing risks. Death without relapse was considered a competing risk for relapse. Relapse was considered a competing risk for TRM. Death without count recovery was considered a competing risk for count recovery. Graft failure, relapse, or death without GvHD were considered competing risks for GvHD. Multivariable Cox proportional hazards models were used to assess the effect of DSA status on OS and RFS, while adjusting the potential confounding effects of other covariates by a backward selection procedure. All tests were two-sided and significance was set at a p-value of 0.05. All the analyses were performed using SAS 9.4 (SAS Institutes, Cary, NC).

## Results

We retrospectively identified 304 patients (DSA negative: 274, DSA positive: 30) for study inclusion. Twenty-four patients transplanted prior to 2015 were excluded due to lack of DSA testing. Baseline demographics are presented in Table 1. The majority of patients with DSAs (in both untreated and treated categories) were women (n = 27). More than half of all patients had a diagnosis of AML. Twenty-one percent of patients who had no DSAs and 31% of untreated DSAs patients had prior allo-HCT, whereas none of the treated DSA patients had prior allo-HCT (p = 0.092). DRI was not uniformly distributed among the cohorts; 50% of patients in the control group and 44% of patients with DSAs who were untreated were classified as Very High/High versus 14% patients with treated DSAs (p = 0.004). The majority (78%) of patients with treated DSAs were intermediate risk by DRI.

Patients with DSAs had a median of two DSAs (range: 1–5). Forty-seven percent had DSAs to class I HLA alone, 23% to class II alone and 30% to both classes. Median MFI of strongest DSA was 9260 (range: 2065–27085). The strongest DSA was to class I in 17 of 30 cases and class II in 13 of 30 cases (Table 2).

**Figure 2:**
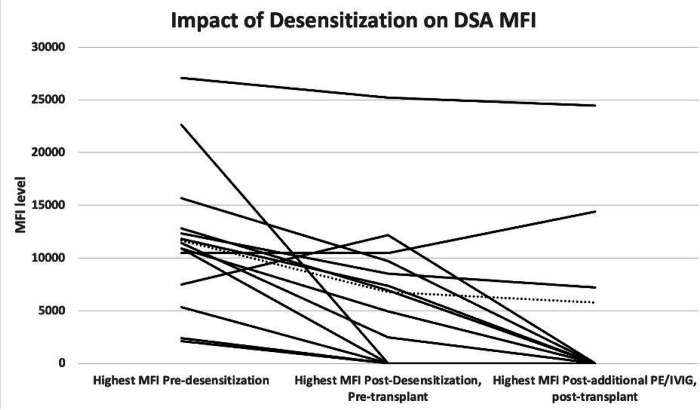
MFI (Mean Fluorescence Intensity) value of the highest DSA (Donor specific antibody) at three time points including pre-desensitization, post-desensitization pre-transplant (Day −1), and post additional desensitization PE/IVIG post-transplant (Day +2). Each line represents 1 of 14 patients with DSAs who went through the desensitization protocol. Average MFI of all patients at these time points are illustrated via dashed line.

Fourteen patients with DSAs underwent desensitization. They received a median of 6 sessions of plasmapheresis and 3 doses of IVIg. Nine out of fourteen patients received additional session of plasmapheresis after transplant. One patient received rituximab as part of desensitization, see patient #13 in desensitized group in Table 2. Of the 14 patients with DSAs, highest DSA MFI decreased in 11 (79%) by a median of 5418 MFI (range: 1436–15,667 MFI). The median MFI after treatment was 2457. Four treated patients (29%) cleared all DSAs prior to transplant (Day −1) and did not undergo additional plasmapheresis/IVIG sessions. Nine patients (64%) had elevated DSAs immediately prior to transplant and underwent additional desensitization on days + 1 and + 2. Four of the nine cleared all DSAs by day + 2 (Fig. 2). In total, 8 of 14 (57%) patients cleared all DSAs by day + 2 or earlier.

**Figure 3:**
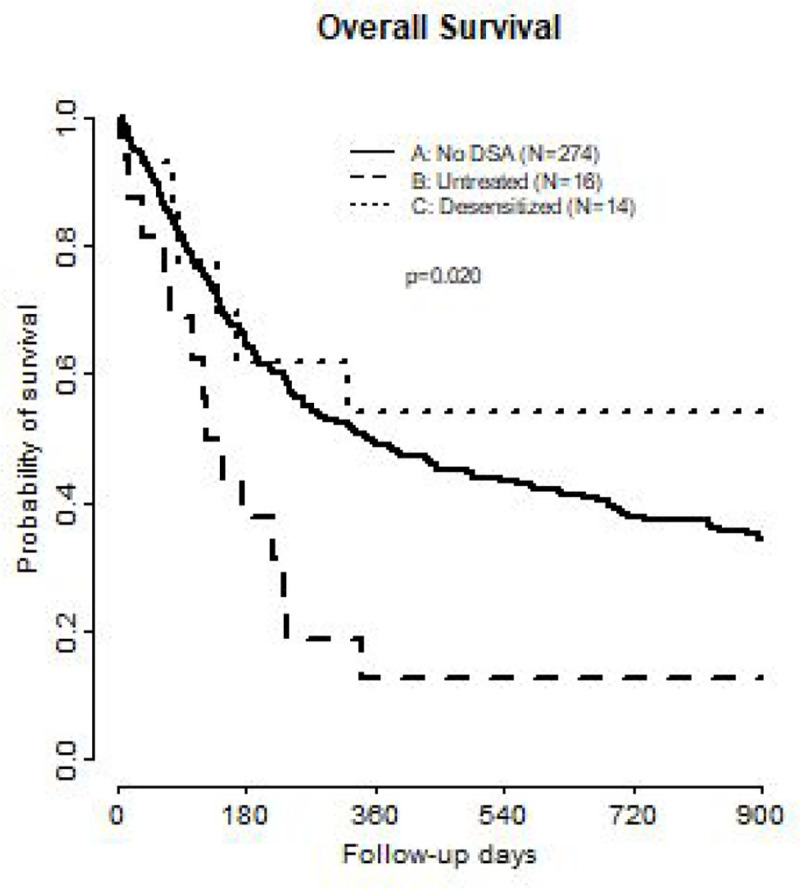
Overall Survival comparison of patients with no DSA, untreated patients with DSAs, and desensitized patients with DSAs. DSA Donor Specific Antibody.

**Figure 4:**
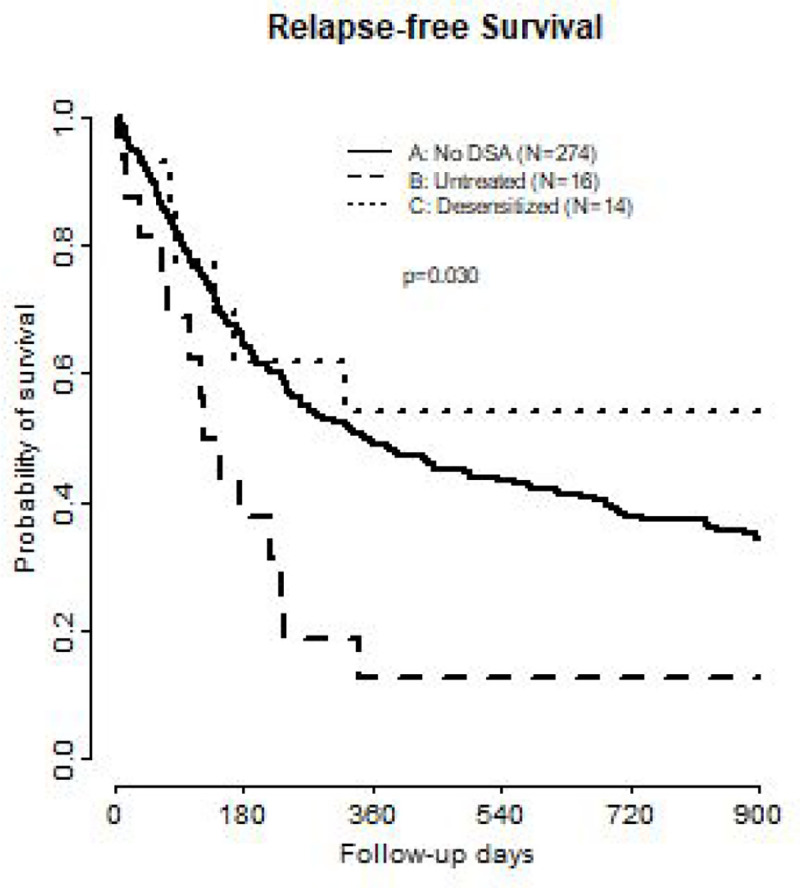
Relapse Free Survival comparison of patients with no DSAs, untreated patients with DSAs, and desensitized patients with DSAs. DSA Donor Specific Antibody. RFS Relapse Free Survival.

OS was significantly different between the groups and, more specifically, OS was worse in patients with untreated DSAs. There were similarly positive results between treated DSA and patients without DSA (OS median: control: 352 days vs. treated: 1331 days vs. untreated: 137 days, p = 0.02, Fig. 3). RFS was also significantly different between the groups however with similar RFS in treated DSA and control groups (RFS median: control: 248 vs. treated: 322 v. untreated: 119, p = 0.03) (Fig. 4). The cumulative incidence of NRM was similar when comparing desensitized patients to control (HR: 1.41, 95% CI 0.62–3.20, p = 0.41), while NRM was significantly higher in untreated DSA compared to control (HR 2.05, 95% CI 1.01–4.16, p = 0.048). The cumulative incidence of relapse for the untreated DSA group (HR: 0.86, 95% CI 0.33–2.24) and treated DSA group (HR: 0.34, 95% CI 0.09–1.32) was not different from the control group. The RFS was significantly different between the groups (treated DSA vs. untreated DSA) while the cumulative incidence of relapse was similar across all groups. The hazard ratios between the two groups show that the treated DSA group had less cumulative incidence of relapse at 0.34 compared to 0.86 in the untreated group. These results are not statistically significant as our cohorts are small but could suggest a consistency between RFS and cumulative incidence in relapse.

Due to significant concern for confounding in this analysis, we constructed a multivariable model to adjust for imbalances in patient and clinical characteristics. Our model included disease risk index (Very High/High, Intermediate, Low), performance status (< 90), HCT-CI score (classified as 0–1, 2, 3, ≥ 4), and prior allogeneic HCT. After MVA, presence of an untreated DSA was still associated with significantly lower OS when compared to the control group (HR 1.80, 95% CI 1.01–3.21, p = 0.046). Survival was not adversely affected by undergoing transplant with a DSA after desensitization when compared to patients without DSAs (HR: 0.69, 95% CI 0.30–1.59, p = 0.37).

**Figure 5:**
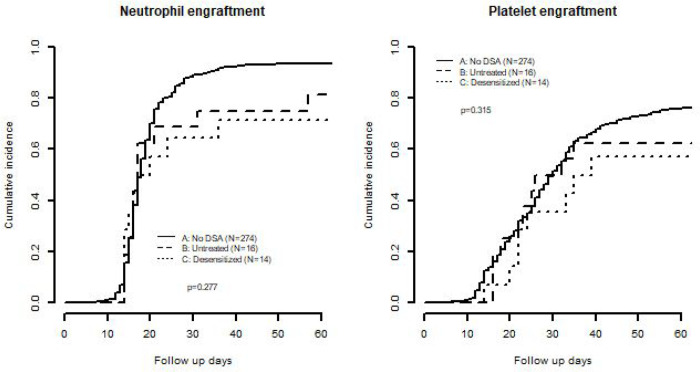
Left. Cumulative incidence of neutrophil engraftment and Right. Cumulative incidence of platelet engraftment between the three groups of patients: patients with no DSA, untreated patients with DSAs, and desensitized patients with DSAs.

There was no difference in the cumulative incidence of neutrophil or platelet engraftment between the groups (Fig. 5). However, a numerically higher number of patients with no DSAs had neutrophil and platelet engraftment (94%, 81% respectively) when compared to the treated DSA group (79%, 64% respectively) and the untreated DSA group (81%, 63% respectively). Regarding graft failure, we do not see any significant difference between the control group (20/274 with graft failure, 7%), treated DSA group (2/14 patients with graft failure, 14%), and the untreated DSA group (with 1/16 with graft failure, 6%). There was no difference in delayed neutrophil engraftment (defined as time to neutrophil engraftment ≥28 days) between the control group (21/274, 8%), treated DSA group (4/14 patients, 29%), and the untreated DSA group (with 3/16, 19%).

## Discussion

In this study, we presented data regarding the impact of a desensitization protocol with a backbone of plasmapheresis and immunoglobulin. We found this protocol lowers DSA levels, as measured by MFI, in most patients. Patients with DSAs undergoing haplo-HCT after this desensitization protocol had similar overall survival and relapse-free survival to those patients who did not have DSAs. Though limited by small number, these results were significant in a multi-variable analysis adjusting for potential confounders. The above results support our desensitization protocol in patients who require haplo-HCT with DSAs to their only suitable donor.

The analysis presented in this paper builds on our prior work regarding haplo-HCT patients with DSAs. A previous report from our center showed a striking decrease in overall survival for patients with untreated DSAs (HR: 4.65, 95% CI: 1.98–10.90) (5). This was consistent with another study of patients undergoing haplo-HCT showed that quantitative levels of DSA were associated with increased poor graft function (PGF) and TRM, with decreased OS (20). A recent meta-analysis by Huang and colleagues also showed a strong association of DSAs with poor transplant-related outcomes, including graft failure, OS and PFS, though it was unable to assess the impact of desensitization treatment due to limited data (21). Our study and many others have shown the presence of DSAs lead to poor transplant-related outcomes. If haplo-transplant with DSAs is the only option for a patient, it is important to decrease DSAs through a desensitization protocol. Our study demonstrates a desensitization protocol that effectively reduces DSAs and leads to similar OS and RFS when compared to those donors without DSAs.

When considering haploidentical donors, DSAs should be tested when typing potential donors. We recommend testing DSAs again within 30 days of haplo-HCT, as MFI levels can change, and other antibodies may be identified. Our study also supports DSA testing after desensitization but prior to transplant in order to guide the addition of further desensitization sessions on days + 1 and + 2. The decision to pursue haploidentical donor transplant should be deliberated via multidisciplinary teams to assess feasibility. Desensitization takes significant planning as plasmapheresis normally requires specialized staff and must begin weeks prior to transplant.

Although there is paucity of data regarding specific methods of desensitization, an early report by Gladstone and colleagues showed reduction of DSA to negative or weak levels in 8 out of 9 patients (9). The aforementioned protocol is based on the practice developed for renal transplant candidates at the Johns Hopkins Comprehensive Transplant Center, which includes alternate-day, single-volume plasmapheresis with anti-CMV hyper-intravenous immunoglobulin (IVIg) (100 mg/kg), tacrolimus (1 mg, IV per day), and mycophenolate mofetil (1 g, twice daily) starting 1 to 2 weeks before the beginning of BMT conditioning, depending upon each patient’s starting DSA levels. Our protocol showed a decreased MFI in 79% of patients, with only 59% of patients achieving complete clearance on Day + 2 after transplant. The importance of complete DSA clearance versus DSA reduction is an important scientific question and should be addressed in future studies using larger cohorts. The significance in achieving clearance before or after transplant is another valid question that should be addressed.

Regarding the optimal desensitization protocol and agents, a variety of innovative approaches are being explored; one study using IVIG and bortezomib reported reduction of MFI to < 2000 in only 2/14 patients. They subsequently used plasmapheresis, rituximab, and IVIG and achieved MFI < 2000 in 12/14 patients (22). A more recent study using two sessions of double-filter plasmapheresis (Day −7, −5) in the week prior to transplant in combination with rituximab on day −4 shows significantly decreased DSAs prior to transplant (L. Liu et al., 2023). Another study reported numerically similar OS and engraftment rates using the standard backbone of plasmapheresis and IVIG combined with buffy coat and rituximab when compared to patients without DSAs (23). A fourth study reported statistically significant improvements in graft function and numerical improvements in NRM and OS with a single dose of rituximab in patients with DSA (versus untreated patients) (24). However, significant heterogeneity exists, even within high volume transplant centers. Additional prospective trials of novel agents are needed to define the optimal approach.

Our study has notable limitations. First, our cohort of treated patients is notably small, with only 14 patients undergoing desensitization. Our desensitization protocol for these 14 patients has the same backbone of plasmapheresis and IVIg, however, the number of plasmapheresis sessions and IVIG is not pre-specified and was left to attending discretion. It is important to note when stratifying by disease risk index, the proportion of high-risk patients is higher in the untreated group, which could suggest selection bias. However, it is important to note untreated DSA patient group was retrospectively identified. In our study, the date of transplant for most untreated DSA patients is from prior to 2015, when desensitization for DSAs was not commonly performed; and if DSAs were present they were between 2 000 and 4 000 MFI, suggesting lower DSA burden and lower risk transplant. There is also a chance of misclassification of patients’ DSA status due to rare HLA alleles not represented in our SAB panel, as outlined in the methods section. Another limitation is that our study is retrospective and non-randomized. The use of DSA-positive donor-recipient pairs is generally avoided at our center, even with desensitization protocols in place. We also acknowledge there is a high number of patients in the untreated cohort who have had prior allogeneic transplant, which suggests higher risk disease and could affect TRM and OS. While we observed a significantly improved OS between desensitized and non-desensitized patients with DSAs, this may be attributable to other factors in addition to our desensitization protocol, such as a higher risk disease in non-desensitized group. These two aforementioned groups were also transplanted in different eras with the desensitized group having access to improved transplant-related care and interventions. Consequently, a selection bias is almost certainly present in our analysis. We performed a MVA to adjust for known confounders, but some residual cofounding may exist. Larger, multi-institutional studies are needed to determine an optimal desensitization protocol. These studies should be adequately powered to examine many questions which we are unable to address in our study, including the optimal level of DSA reduction by MFI and the utility of adding novel agents such as rituximab, bortezomib, daratumumab or buffy coat in haplo-HCT patients with DSAs.

## Conclusion

Here we report our institutional experience desensitizing patients with DSAs undergoing haplo-HCT. We compare outcomes with patients without DSAs and patients with DSAs who did not undergo desensitization. Our data shows that treatment of DSAs prior to transplant with a structured desensitization protocol may produce outcomes equivalent to patients undergoing transplant without DSA. These data support the use of haplo-HCT donors in the presence of DSAs if 1) no other donor is available, and 2) desensitization with plasmapheresis and immunoglobulin is undertaken. Desensitization should be planned in advance in order to avoid delays in transplantation. Further studies are needed to define the optimal desensitization protocol and DSA reduction targets for treatment.

## Figures and Tables

**Table 1:** 

* The parametric p-value is calculated by ANOVA for numerical covariates and chi-square
test for categorical covariates.
** The non-parametric p-value is calculated by the Kruskal-Wallis test for
numerical covariates and Fisher’s exact test for categorical covariates.
*** Disease Risk Index
+ Hematopoietic Cell Transplant Comorbidity Index
++ Myeloablative chemotherapy (A false answer means Reduced Intensity Chemotherapy or RIC)
+++ Karnofsky Performance Status
